# Transition metal doped CeO_2_ for photocatalytic removal of 2-chlorophenol in the exposure of indoor white light and antifungal activity

**DOI:** 10.3389/fchem.2023.1126171

**Published:** 2023-04-19

**Authors:** M. Tariq Qamar, Shahid Iqbal, M. Aslam, Ahmad Alhujaily, Anum Bilal, Komal Rizwan, Hafiz Muhammad Umer Farooq, Tahir Ali Sheikh, Ali Bahadur, Nasser S. Awwad, Hala A. Ibrahium, Rasmiah S. Almufarij, Eslam B. Elkaeed

**Affiliations:** ^1^ Department of Chemistry, Forman Christian College (A Chartered University), Lahore, Pakistan; ^2^ Department of Chemistry, School of Natural Sciences (SNS), National University of Science and Technology (NUST), Islamabad, Pakistan; ^3^ Centre of Excellence in Environmental Studies (CEES), King Abdulaziz University, Jeddah, Saudi Arabia; ^4^ Biology Department, College of Science, Taibah University, Al Madinah Al Munawarah, Saudi Arabia; ^5^ Department of Chemistry, University of Sahiwal, Sahiwal, Pakistan; ^6^ Department of Chemistry, Government Islamia College, Civil Lines, Lahore, Pakistan; ^7^ Department of Chemistry, The Islamia University of Bahawalpur, Bahawalpur, Pakistan; ^8^ Department of Chemistry, College of Science and Technology, Wenzhou-Kean University, Wenzhou, China; ^9^ Chemistry Department, Faculty of Science, King Khalid University, Abha, Saudi Arabia; ^10^ Biology Department, Faculty of Science, King Khalid University, Abha, Saudi Arabia; ^11^ Department of Semi Pilot Plant, Nuclear Materials Authority, El Maadi, Egypt; ^12^ Department of Chemistry, College of Science, Princess Nourah Bint Abdulrahman University, Riyadh, Saudi Arabia; ^13^ Department of Pharmaceutical Sciences, College of Pharmacy, AlMaarefa University, Riyadh, Saudi Arabia

**Keywords:** modified CeO_2_, band gap energy, photocatalytic removal, 2-chlorophenol, nanocompoiste, antifungal activity

## Abstract

Besides natural sunlight and expensive artificial lights, economical indoor white light can play a significant role in activating a catalyst for photocatalytic removal of organic toxins from contaminated water. In the current effort, CeO_2_ has been modified with Ni, Cu, and Fe through doping methodology to study the removal of 2-chlorophenol (2-CP) in the illumination of 70 W indoor LED white light. The absence of additional diffractions due to the dopants and few changes such as reduction in peaks’ height, minor peak shift at 2θ (28.525°) and peaks’ broadening in XRD patterns of modified CeO_2_ verifies the successful doping of CeO_2_. The solid-state absorption spectra revealed higher absorbance of Cu-doped CeO_2_ whereas a lower absorption response was observed for Ni-doped CeO_2_. An interesting observation regarding the lowering of indirect bandgap energy of Fe-doped CeO_2_ (∼2.7 eV) and an increase in Ni-doped CeO_2_ (∼3.0 eV) in comparison to pristine CeO_2_ (∼2.9 eV) was noticed. The process of *e* ^*-*^– *h* ^*+*^ recombination in the synthesized photocatalysts was also investigated through photoluminescence spectroscopy. The photocatalytic studies revealed the greater photocatalytic activity of Fe-doped CeO_2_ with a higher rate (∼3.9 × 10^−3^ min^-1^) among all other materials. Moreover, kinetic studies also revealed the validation of the Langmuir-Hinshelwood kinetic model (R^2^ = 0.9839) while removing 2-CP in the exposure of indoor light with a Fe-doped CeO_2_ photocatalyst. The XPS analysis revealed the existence of Fe^3+^, Cu^2+^ and Ni^2+^ core levels in doped CeO_2_. Using the agar well-diffusion method, the antifungal activity was assessed against the fungus *M. fructicola* and *F. oxysporum*. Compared to CeO_2_, Ni-doped CeO_2_, and Cu-doped CeO_2_ nanoparticles, the Fe-doped CeO_2_ nanoparticles have outstanding antifungal properties.

## Introduction

A major environmental threat that humanity faces today is the contamination of water caused by hazardous chemicals released into the environment by industrial processes. Routine daily activities can cause hazardous materials to be introduced into natural water resources, such as aquifers, lakes, oceans, rivers, and groundwater aquifers. Continually introducing such contaminants into natural water can result in an alteration of the characteristic features of natural water as well as polluted water. This is extremely unsuitable for human health. Chemical pollutants like dyes for textiles, halogenated compounds, substituted phenols, insecticides, pesticides, weed killers, and herbicides are known to be carcinogenic and pose major health risks to humans (Anku et al., 2017; Samanta et al., 2019; Brillas and Garcia-Segura, 2020; Kanan et al., 2020; Rafiq et al., 2021). Among toxic organic pollutants- chlorophenols and nitrophenols, because of their stable structure, toxic and carcinogenic effects on the human being, have been a remaining area under concern (Chiou et al., 2008; Adewuyi et al., 2016; Umukoro et al., 2017; Sharma et al., 2019; Barakat et al., 2020). Therefore, it is highly desirable to remove these toxins effectively from the polluted water.

The structural properties of these phenols and their derivatives have made them difficult to remove from the polluted water by classical methods, i.e., activated carbon adsorption, chemical oxidation, and biological treatment. Because activated carbon adsorption leads to phase separation without degradation of injurious pollutants, the chemical oxidation method is unable to remove pollutants efficiently, and biological treatments are slow and pH and temperature dependent, while the advanced oxidation process seems to be better for effective removal of such sort of destructive pollutants using light (Eryılmaz and Genç, 2021; Ren et al., 2021; Saputera et al., 2021).

Advanced oxidation leads to the formation of highly reactive species which ultimately react with organic pollutants to degrade them successfully (Mazivila et al., 2019; Du and Zhou, 2021; Gallo-Cordova et al., 2021; Huang et al., 2021; Luo et al., 2021). Among advanced oxidation processes, photocatalytic degradation and mineralization are efficient methods of converting highly toxic organic pollutants to harmless species under ambient conditions using light and a photocatalyst through homogeneous or heterogenous photocatalysis. In addition, heterogeneous photocatalysis is considered advantageous over homogenous photocatalysis due to an easy retrievability of a catalyst from the reaction mixture and then the reusability of a catalyst (Gisbertz and Pieber, 2020). Following the concept of the advanced oxidation process, the heterogeneous photocatalytic reaction starts with the generation of H_2_O_2_, OH^∙^, O_2_^∙-^, HOO^∙^ and H^+^ species by the absorption of a photon having energy equal to or greater than the band gap energy of the photocatalyst which is being used (Loeb et al., 2019; Pandey et al., 2020; Serrà and Philippe, 2020; Danish et al., 2021). In addition, hydroxyl and superoxide anion radicals react with phenols, depending on the position and nature of substituent groups on ph enols, to degrade them into some oxygenate intermediates and consequently, these oxygenates mineralize to their respective harmless species (Qamar et al., 2017a; Qamar et al., 2017b; Alhogbi et al., 2020).

Metal oxide-assisted photocatalytic wastewater treatment is a relatively prospective subject and growing rapidly to remove hazardous pollutants from contaminated water. In this context, metallic oxide semiconductors such as TiO_2_, ZnO, Fe_2_O_3_, SnO_2_, WO_3_, Bi_2_O_3_, V_2_O_5_, Cu_2_O, NiO, etc. have been studied extensively both in artificial and natural light sources to acquire pollution free water (Ahmed et al., 2010; Oturan and Aaron, 2014; Wang et al., 2014; Qamar et al., 2015; Zangeneh et al., 2015; Aslam et al., 2018). However, exploration of a potential photocatalyst for the effective removal of toxic pollutants from the wastewater has not over yet and researchers are paying attention to study the photocatalytic properties of CeO_2_-based photocatalysts for the abatement of organic pollutants. Although, bare CeO_2_ has wide application in electrocatalysis, solar cells, fuel cells and photocatalysis due to its high chemical stability, low toxicity and greater oxygen storage capacity (Ma et al., 2019; Fauzi et al., 2022). However, its performance in photocatalysis is unsatisfactory due to greater photo excitons’ recombination, lower absorption cross-section of light spectrum and higher band gap (2.8–3.1 eV) energy (Ma et al., 2019; Fauzi et al., 2022). Therefore, it is a need to modify CeO_2_ in order to increase its utilization as a photocatalyst for removal of organic toxins. The unique thing of this study is the introduction of transition metals into CeO_2_ without significantly changing the cubic structure of CeO_2_ photocatalyst for the removal of 15 ppm 2-chlorophenol in the illumination of 70 W indoor white light.

Previously, researchers utilized expensive artificial light sources such as solar simulator, Hg and Xe lamps for photocatalytic degradation of various organic toxins using variety of photocatalysts (Tang et al., 2004; Hao et al., 2015; Aslam et al., 2016; Xu et al., 2017). In addition, a few studies are available for the photocatalytic removal of 2-CP using CeO_2_-based photocatalysts such as CeO_2_, g-C_3_N_4_(0.94)/CeO_2_(0.05)/Fe_3_O_4_(0.01), TiO_2_-CeO_2_-ZrO_2_ (Aslam et al., 2016; García-Hernández et al., 2019; Rashid et al., 2019). However, various combinations of CeO_2_ with metals, non-metals and photocatalysts such as Ag_2_CO_3_, rGO, CdS, AgBr, SrFe_12_O_9_, BiOCl, g-C_3_N_4_, Co_3_O_4_, etc., are reported for the photocatalytic removal of organic dyes, phenols and pharmaceutical ingredients (Ma et al., 2019; Yao et al., 2021; Fauzi et al., 2022). Moreover, literature reveals that a photocatalysis setup while using an economical 70 W indoor white light for the removal of 2-CP over the proposed composition of CeO_2_-based photocatalysts has not been reported before.

In this study, CeO_2_ has been synthesized using a co-precipitation methodology and its modification was executed through doping with transition metals, i.e., Fe, Ni and Cu because 3d transition metals are considered capable dopants in tuning the properties of CeO_2_ for catalysis applications (Yue and Zhang, 2009; Elias et al., 2014; Qi et al., 2019). The spectral response and bandgap of the synthesized materials were evaluated using UV-visible diffuse reflectance spectroscopy (UV-visible DRS) whereas photoluminescence (PL) fluorometer was used to investigate *e* ^*-*^–*h* ^*+*^ recombination. X-ray diffraction (XRD) and X-ray photoelectron spectroscopy (XPS) were used to evaluate the structural and chemical characterization of synthesized materials. The photocatalytic activity of the synthesized photocatalysts was studied for the removal of 2-chlorophenol (2-CP) the kinetics for the photodegradation of 2-CP were also investigated.

## Experimental

### Materials

Cerium (III) nitrate hexahydrate [Ce(NO_3_)_3_.6H_2_O, Sigma-Aldrich, ≥99%], iron (III) nitrate non-ahydrate [Fe(NO_3_)_3_.9H_2_O, Sigma-Aldrich, 99.95%], nickel (II) nitrate hexahydrate [Ni(NO_3_)_2_.6H_2_O, Sigma-Aldrich, ≥99%], copper (II) nitrate trihydrate [Cu(NO_3_)_2_.3H_2_O, Sigma-Aldrich, 99.99%], ethanol (C_2_H_5_OH, Sigma-Aldrich, ≥99.8%), nitric acid [HNO_3_, Sigma-Aldrich, 70%], acetone [CH_3_COCH_3_, Sigma-Aldrich], potassium hydroxide [KOH, Sigma-Aldrich, 99.5%] and triton X-100 [Tx-100, Sigma-Aldrich] were used without further purification for the synthesis of photocatalysts.

### Synthesis of pristine CeO_2_

In a typical synthesis of CeO_2_, 30 g Ce(NO_3_)_3_.6H_2_O was dissolved in 100 mL distilled water with continuous stirring until the formation of a clear solution. After complete dissolution, 3 mL Triton X-100 was added to the clear solution under continuous stirring for 30 min at room temperature. To hydrolyze the solution, 0.1M potassium hydroxide (KOH) was added to the solution dropwise till the formation of yellow precipitate near pH 9 at 50°C. The mixture containing the yellow precipitate, surfactant and KOH content was washed several times with distilled water and then with ethanol/water (30:60) mixture to remove surfactant and basic contents present in the mixture until the mixture attained the neutral pH. The precipitate was separated from the reaction mixture through filtration and the obtained yellow precipitate was again washed with ethanol/water (30:60) mixture and dried overnight at 100°C in a vacuum oven and then calcined in a muffle furnace for 4 h at 400°C. The calcined material was ground using mortar and pestle to get fine powder of CeO_2_ photocatalyst.

### Synthesis of modified CeO_2_

For the synthesis of Cu doped CeO_2_, 0.381 g Cu(NO_3_)_2_.3H_2_O and 30.681 g Ce(NO_3_)_3_.6H_2_O were dissolved separately in distilled water with continuous stirring at room temperature till the formation of clear solutions and marked solution A and B, respectively. Then, solution A was added slowly into the beaker having solution B with continuous stirring at 50°C for 10 min followed by the addition of 3 mL Triton X-100 under continuous stirring for 30 min. The mixture of both precursors and surfactant was then hydrolyzed with the slow addition of 0.1M KOH till the formation of precipitate at pH 9. The formed precipitate was separated through filtration, washed with water and ethanol/water (30:60) mixture, and dried overnight in a vacuum oven at 100 °C. The dried sample was then subjected to the muffle furnace for calcination at 400°C for 4 h. The calcined material was ground using mortar and pestle to get fine powder of Cu doped CeO_2_ photocatalyst. The same procedure was applied for the synthesis of Ni-doped CeO_2_ and Fe-doped CeO_2_ except for the different amounts of precursors, e.g., 0.496 g Ni(NO_3_)_2_.6H_2_O and 0.723 g Fe(NO_3_)_3_.9H_2_O about the Ni and Fe contents, respectively for 30.681 g of Ce(NO_3_)_3_.6H_2_O. The synthesized photocatalytic materials were characterized by UV- visible diffuse reflectance spectroscopy (UV-Vis DRS), photoluminescence (PL), X-ray Diffraction (XRD), scanning electron microscopy (SEM) and X-ray photoelectron spectroscopy (XPS).

### Photocatalytic studies

The photocatalytic performance of transition metal doped CeO_2_ was studied for the removal of 15 ppm 2-chlorophenol under the illumination of 70 W indoor white light (800 × 10^2^ lx) at room temperature. In this context, the synthetic wastewater containing 15 ppm 2-chlorophenol was prepared in the laboratory, and the dose of the photocatalyst was optimized by exposing the suspension having 100 mL of pollutant with varying amounts of CeO_2_ photocatalyst (50, 100, 150, and 200 mg) in a glass reactor of 14 cm (diameter) and 2 cm (height) to the indoor white light for 3 h and the samples were collected and filtered using 0.20 µm syringe filter. The filtered samples were then subjected to UV-visible spectrophotometer for the monitoring of photodegradation of 2-CP and 100 mg of the photocatalyst was found to be the optimum dose of catalyst in 100 mL of pollutant. The optimized amount, i.e., 100 mg/100 mL of all four photocatalysts was further used for the evaluation of their photocatalytic degradation efficiency in removing 2-CP under the exposure of 70 W indoor LED white light (800 × 10^2^ lx, 400–800 nm, Opple Lighting Co., Ltd. China) at room temperature and samples were collected after 30, 60, 90, 120, 180 and 240 min and filter using 0.20 µm syringe filter for UV-visible spectrophotometric analysis to evaluate the kinetic of photodegradation.

To study the removal of the target pollutant from the polluted water, the samples were collected using a syringe filter from the suspension exposed to the light after a regular interval and were subjected to UV-visible spectrophotometer for the determination of the concentration of the removed pollutant which ultimately led to the % removal of target pollutant by the photocatalyst under the exposure of light using the following equation.

$$\%\;removal=\frac{C_{o}-C_{t}}{C_{o}}\times 100$$ (1)

Here, *C*_*o*_ is the pollutant initial concentration whereas *C*_*t*_ is the concentration of the pollutant after exposure time “*t*.” Moreover, the validity of Langmuir Hinshelwood (L-H) kinetic models was also studied using the following equation to evaluate the kinetics of photocatalytic removal of organic pollutants under exposure to light.

$$\ln\frac{C_{o}}{C_{t}}=k\times t$$ (2)

Here, *k* is the rate constant which was determined from the slope by plotting *ln* $\frac{C_{o}}{C_{t}}$ Vs. *t*.

## Results and discussion

**FIGURE 1:**
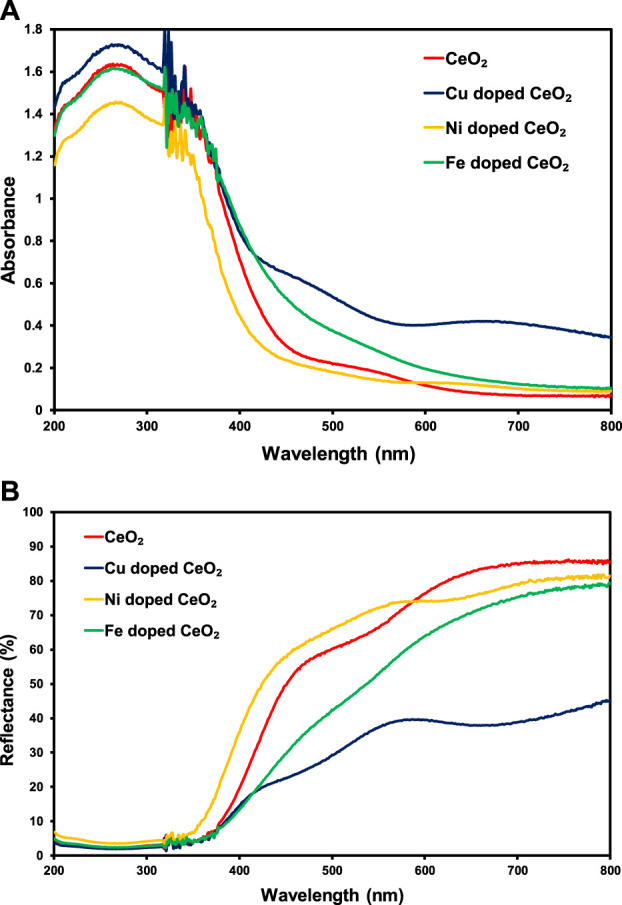
The comparison of solid-state **(A)** absorption and **(B)** reflectance (%) spectra of pristine CeO_2_ and doped CeO_2_.

The solid-state absorption response of the pristine CeO_2_ compared to the modified CeO_2_ is presented in Figure 1A in UV (200–400 nm) as well as visible (400–800 nm) regions of the spectrum. Wherein, Cu-doped CeO_2_ shows higher absorbance in both UV and visible regions than other synthesized materials. Interestingly, Cu-doped CeO_2_ and Fe-doped CeO_2_ showed higher absorbance of visible light than CeO_2_ and Ni-doped CeO_2_ whereas in the UV region of the spectrum only Cu-doped CeO_2_ presented higher absorbance than other photocatalysts. So, the insertion of Cu and Fe content into the structure of CeO_2_ leads to an increase in the spectral response of CeO_2_ whereas the same amount of Ni content has a detrimental effect on the spectral response of the pristine CeO_2_. Moreover, the response of these photocatalysts in reflecting the light spectrum has also been explored in Figure 1B. Wherein, lower reflectance (%) was noticed for CeO_2_ having copper contents followed by Fe and Ni contents in the lower energy region of the spectrum. This higher absorbance and lower reflectance (%) were attributed to the higher absorbance ability of copper content present in the structure of CeO_2_ as compared to Fe and Ni.

**FIGURE 2:**
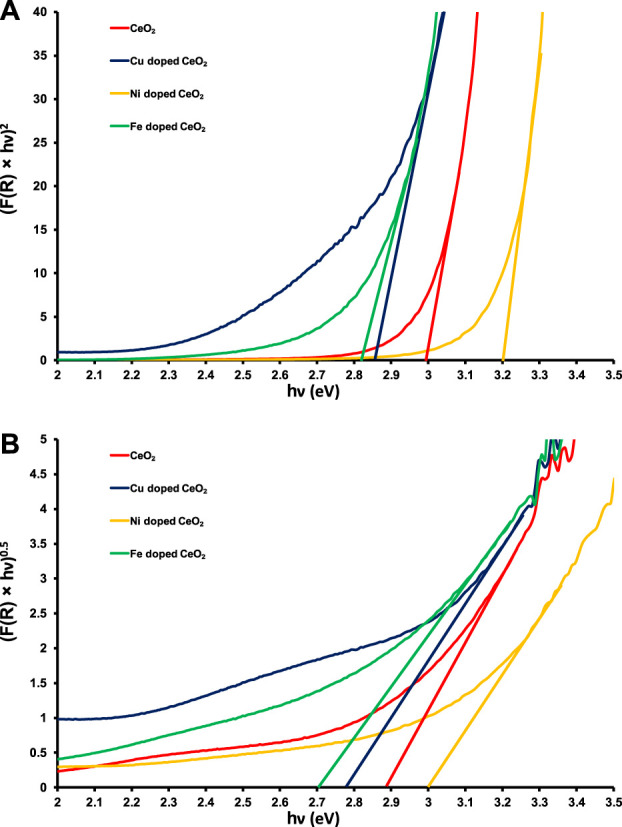
The graphical evaluations of **(A)** direct and **(B)** indirect band gaps of pristine and doped CeO_2_.

The direct and indirect band gap evaluations (Figures 2A, B) reveal a lowering in the band gap energy of Cu and Fe-doped CeO_2_ photocatalysts as compared to pristine CeO_2_ was noticed which may be attributed to the shifting of conduction band edges to lower energy due to the addition of Cu and Fe contents in the structure of CeO_2_ (Yue and Zhang, 2009; Qi et al., 2019). Whereas a significant increase in the band gap energy of Ni-doped CeO_2_ was observed. The direct and indirect band gaps of the pristine CeO_2_ were noticed at ∼3.0 and ∼2.9 eV, respectively which have good agreement with the literature values (Aslam et al., 2016). Moreover, the direct band gap energies found for Cu, Fe, and Ni-doped CeO_2_ photocatalysts are ∼2.85, ∼2.8, and ∼3.2 eV whereas evaluated indirect band gap energies are ∼2.78, ∼2.7, and ∼3.0 eV, respectively as shown in Figures 2A, B. A greater decrease in the band gap energy was noticed for Fe doped CeO_2_ as compared to other photocatalysts which reveal that this photocatalyst requires lower energy as compared to pristine and other modified photocatalysts to make good use of excitons for the removal of organic toxins under the illumination of light.

**FIGURE 3:**
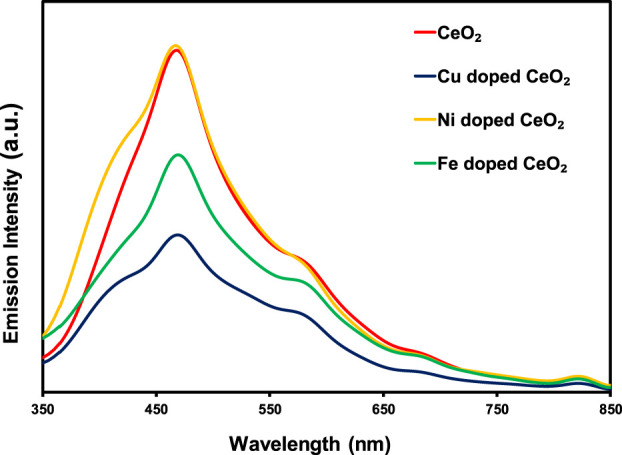
The comparison of PL spectra of pristine CeO_2_ and doped CeO_2_.

Photoluminescence analysis is a promising technique to study the emission intensity of the excited electron under the illumination of light which ultimately leads to the study of *e* ^*-*^–*h* ^*+*^ recombination process. Figure 3 depicts the comparison of photoluminescence spectra of pristine and doped CeO_2_ photocatalysts, wherein a significantly lower emission intensity was noticed for Cu and Ni-doped CeO_2_ as compared to unmodified CeO_2_. The relative decrease in emission intensities of the Cu and Fe doped CeO_2_ as compared to CeO_2_ is strong evidence for lowering *e* ^*-*^–*h* ^*+*^ recombination rate than pristine CeO_2_ which favors the photocatalytic degradation of organic toxins. Moreover, the decrease in strong emission bands around 470 nm is attributed to the trapping of photoexcitons by the surface defects generated due to the insertion of Cu or Fe into the structure of CeO_2_ (George et al., 2020).

**FIGURE 4:**
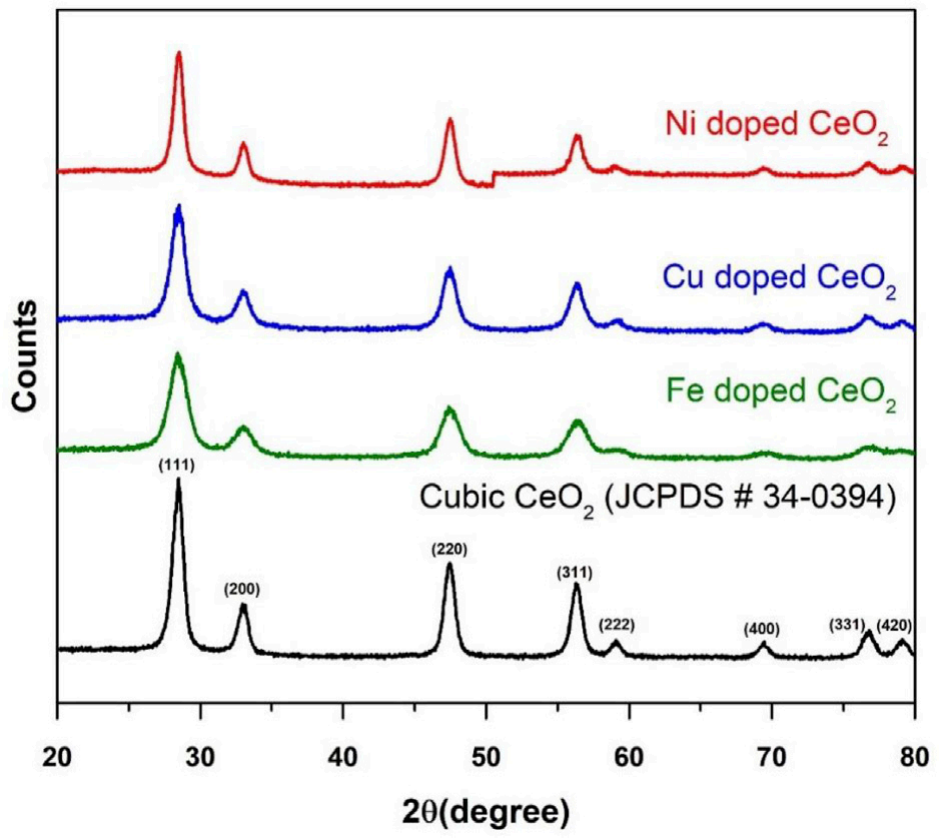
The comparison of XRD patterns of pristine CeO_2_ with doped CeO_2_.

**FIGURE 5:**
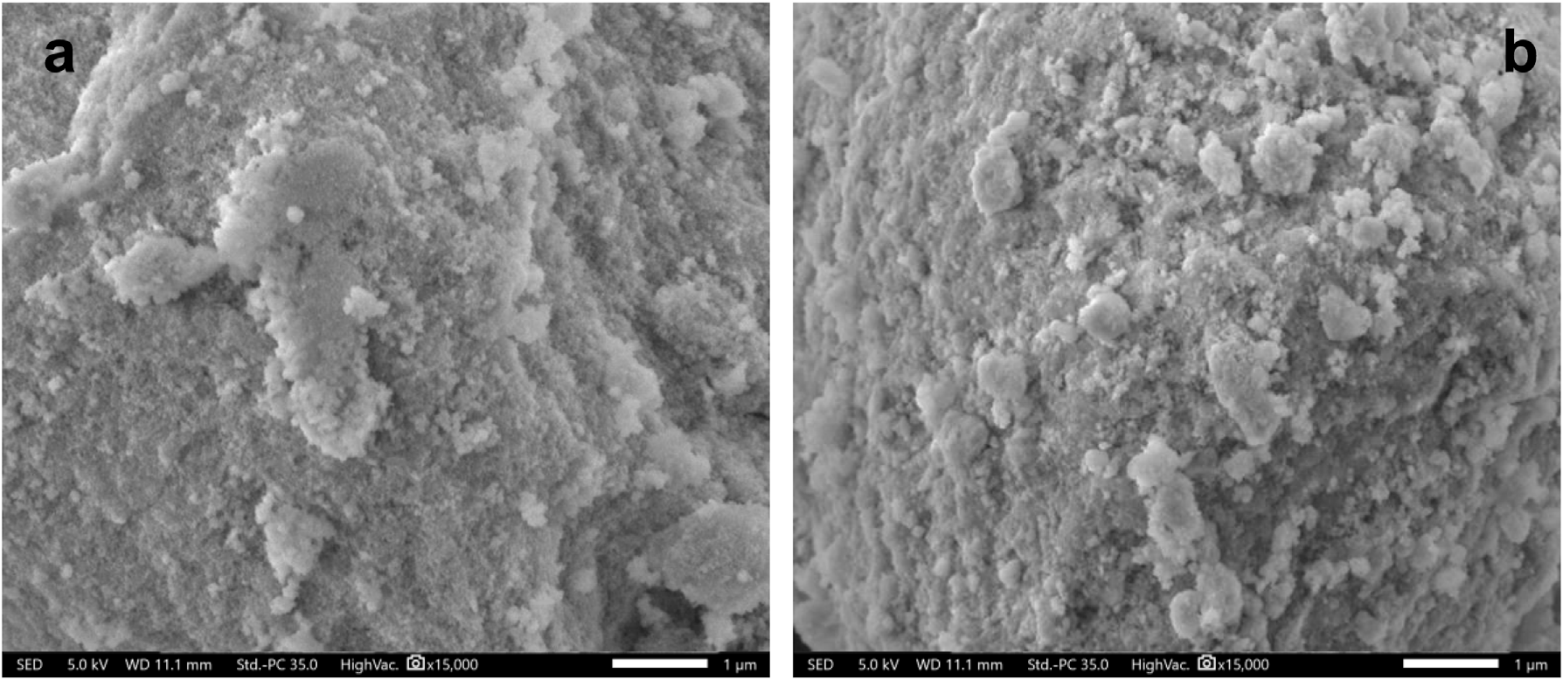
The comparison of scanning electron micrographs of **(A)** pristine CeO_2_ and **(B)** Fe doped CeO_2_.

The comparison of x-ray diffraction patterns of the modified CeO_2_ with pristine CeO_2_ is presented in Figure 4 and the cubic phase of the synthesized CeO_2_ was confirmed by matching diffractions planes (111), (200), (220), (311), (222), (400), (331), and (420) with literature and JCPDS 34-0394 (Aslam et al., 2016). The absence of additional diffraction peaks related to the dopant entities in the respective diffraction patterns verifies the successful doping of CeO_2_ with Cu, Ni, and Fe. Moreover, few changes such as reduction in few peaks’ height, minor peak shift at 2θ (28.525^°^) and peaks’ broadening in XRD patterns of modified CeO_2_ as compared to pristine CeO_2_ also favour the introduction of dopants in CeO_2_ (Kumar et al., 2010). Moreover, the successful insertion of dopants into CeO_2_ without significantly altering its structure were also evident from the micrographs as shown in Figure 5. The average crystallite size of the photocatalytic materials was calculated using a high-intensity diffraction peak at 2θ (28.525^°^) with the help of the Debye-Scherrer equation. The calculated crystallite sizes were 9.76, 3.46, 4.97, and 8.28 nm for pristine CeO_2_, Fe doped CeO_2_, Cu doped CeO_2,_ and Ni doped CeO_2_, respectively. Moreover, a significant difference in doped CeO_2_ as compared to unmodified CeO_2_ were also reported in literature which support the changes in crystallite sizes of Fe and Cu doped CeO_2_ (Yue and Zhang, 2009; Kumar et al., 2010; Qi et al., 2019).

**FIGURE 6:**
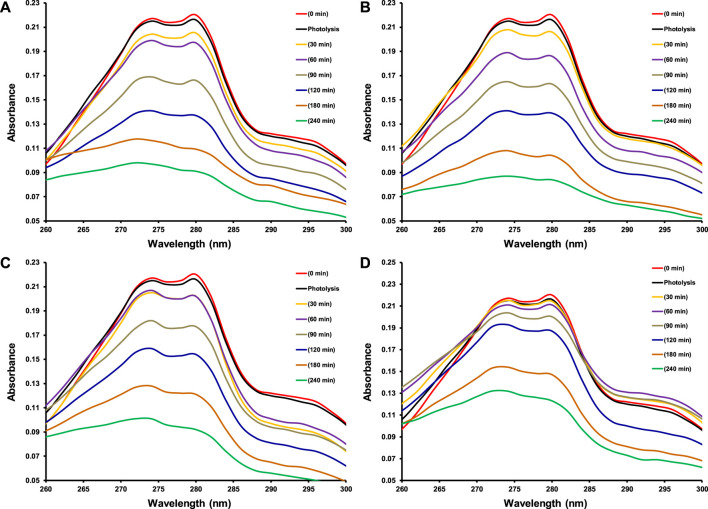
The comparison of absorption spectra for photocatalytic degradation of 15 ppm 2-CP over **(A)** CeO_2_ **(B)** Fe doped CeO_2_ **(C)** Cu doped CeO_2_ and **(D)** Ni doped CeO_2_ at different intervals of time under the illumination of indoor white light (800 × 10^2^ lx).

**FIGURE 7:**
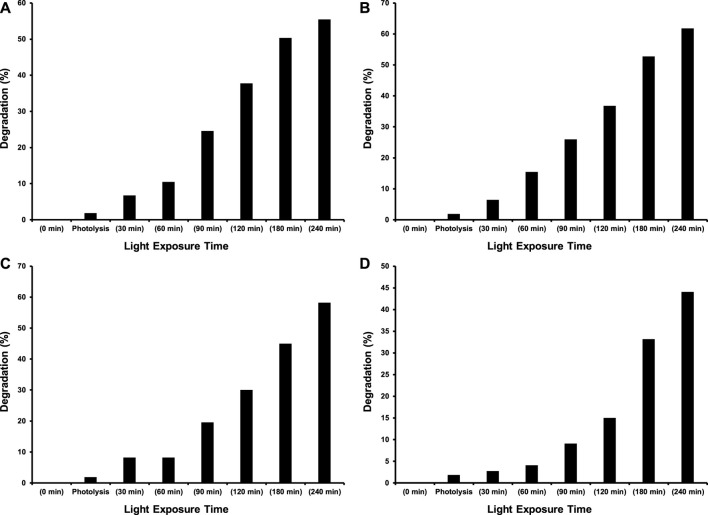
The comparison of % degradation of 15 ppm 2-CP over **(A)** CeO_2_ **(B)** Fe doped CeO_2_ **(C)** Cu doped CeO_2_ and **(D)** Ni doped CeO_2_ at different intervals of time under the illumination of indoor white light (800 × 10^2^ lx).

As this study was designed to investigate the effect of transition metal dopants on the photocatalytic performance of CeO_2_ under the illumination of indoor white light for the degradation of 15 ppm 2−CP. Before the exposure of suspension containing photocatalyst and 2-CP to light as mentioned in the experimental section, the suspension was kept in dark for 30 min to establish an equilibrium between pollutant and catalyst. The photolysis of 2-CP was also evaluated by recording the absorption spectrum of the substrate after 240 min of light exposure without the presence of a photocatalyst. The amount of photocatalyst was also optimized (100 mg of photocatalyst) while studying the photocatalytic removal of 15 ppm 2-CP with varying doses of CeO_2_ catalyst under the illumination of indoor white light. The comparison of absorption spectra of photocatalytic removal of 2-CP over pristine CeO_2_, Fe doped CeO_2_, Cu doped CeO_2_, and Ni-doped CeO_2_ under the illumination of indoor white light (800 × 102 lx) is provided in Figure 6, respectively at different exposure time. Whereas the photocatalytic degradation (%) of 2-CP at different exposure over the pristine and modified CeO_2_ photocatalysts is given in Figure 7, respectively and highest photodegradation (∼ 65%) was noticed for Fe doped CeO_2_ followed by Cu doped CeO_2_ (∼ 60%), pure CeO_2_ (∼59%) and Ni doped CeO_2_ (∼45%) under the exposure of white light after 240 min of exposure. The decreasing trend of removal efficiency (%) of the photocatalysts is given below.

$$Fe\;doped\;{CeO}_{2}\;>\;Cu\;doped\;{CeO}_{2}\;>\;{CeO}_{2}\;>\;Ni\;doped\;{CeO}_{2}$$

**FIGURE 8:**
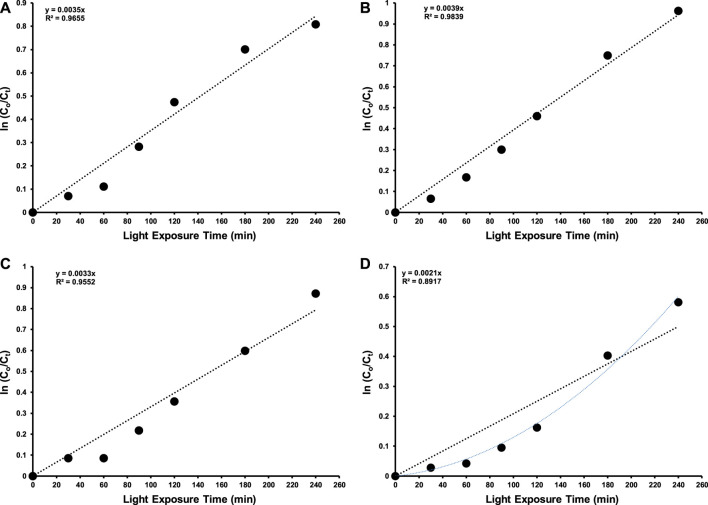
Rate of removal of 15 ppm 2-CP over **(A)** CeO_2_ **(B)** Fe doped CeO_2_ **(C)** Cu doped CeO_2_ and **(D)** Ni doped CeO_2_ at different intervals of time under the illumination of indoor white light (800 × 10^2^ lx).

Moreover, the rate of photodegradation of 15 ppm 2-CP over synthesized photocatalysts was also investigated and higher photocatalytic removal efficiency with rate constant (k = 3.9 × 10^−3^ min^-1^) was observed by Fe doped CeO_2_ than others as shown in Figure 8. The calculated bandgap energy values as shown in Figure 2 also support the possible higher photodegradation efficiency of Fe-doped CeO_2_ due to its lower bandgap energy than CeO_2_. Moreover, a lower photo-excitons’ recombination in PL spectra presented by Fe-doped CeO_2_ also arguments its higher removal efficiency due to the possible charge transferability for the generation of ROS. The decreasing trend of rate of removal of 2-CP by different photocatalysts is provided below.

$$Fe\;doped\;{CeO}_{2}\;>\;{CeO}_{2}\;>\;Cu\;doped\;{CeO}_{2}\;>\;Ni\;doped\;{CeO}_{2}$$

The kinetic study also reveals that the photocatalytic removal of 2-CP by Fe doped CeO_2_, pure CeO_2,_ Cu doped CeO_2_ followed Langmuir Hinshelwood (L-H) kinetic model whereas the photocatalytic removal of 2-CP by Ni doped CeO_2_ did not follow Langmuir Hinshelwood (L-H) kinetic model. Previously, Zhang et al. (2022) reported a decrease in degradation rate of 2-CP removal in the illumination of 5W LED white light as compared to 300 W Xe arc lamp. In another study, BiFeO_3_/Bi_2_Fe_4_O_9_ heterojunctions were able to remove 95% of the 2-CP in the exposure of 150W LED white light (Wang et al., 2022).

**FIGURE 9:**
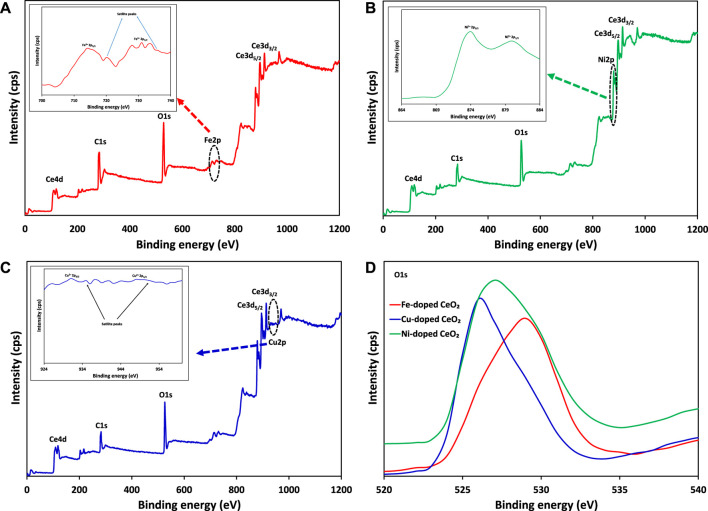
The comparison of XPS survey scan of **(A)** Fe-doped CeO_2_ **(B)** Ni-doped CeO_2_ **(C)** Cu-doped CeO_2_ and **(D)** O1s levels of modified CeO_2_ photocatalysts.

In this study, x-ray photoelectron spectroscopy (XPS) was also carried out to investigate the chemical and electronic states of the elements in modified materials as shown in Figure 9. The presence of Ce3d_3/2_, Ce3d_5/2_, Fe2p_1/2_, Fe2p_3/2_, Cu2p_1/2_, Cu2p_3/2_, Ni2p_1/2_, Ni2p_3/2_ core levels and O1s levels in the synthesized photocatalysts can be seen in Figure 9, which confirm the existence of dopants (Fe^3+^, Cu^2+^ and Ni^2+^) in doped CeO_2_ as shown in inset figures. Moreover, the effect of dopants was also verified by the appearance of peak correspond to O1s level at different binding energies which ultimately confirm the different environment of oxygen.

### Antifungal activity

Using the agar well-diffusion method and amphotericin B as a reference, CeO_2_, Fe-doped CeO_2_, Ni-doped CeO_2_, and Cu-doped CeO_2_ nanoparticles were further assessed for their antifungal activity against *M. fructicola* and *F. oxysporum*. Table 1 provides a summary of the outcomes. According to the antifungal activity data (Table 1), Fe-doped CeO_2_ nanoparticles exhibit greater toxicity when compared to CeO_2_, Ni-doped CeO_2_, and Cu-doped CeO_2_ nanoparticles, with zone inhibition values of 25.3 and 23.1 mm. Due to their small particle size, the Fe-doped CeO_2_ nanoparticles easily pass through the fungal cell membrane, attach to functional protein groups as well as substances that contain phosphorus and sulphur, including DNA, and ultimately result in fungal cell death. The improved antifungal impact was caused by the synergistic interaction of Fe-doped CeO_2_ nanoparticles and decreased size.

**TABLE 1:** Agar Well cut diffusion method zone of inhibition for the antifungal action of CeO_2_, Fe-doped CeO_2_, Ni-doped CeO_2_ and Cu-doped CeO_2_.

Antifungal performance			
Bacterial strains	Samples	Blank	Zone of inhibition (mm)
*M. fructicola*	CeO_2_	0	17.4
	Fe-doped CeO_2_	0	25.3
	Cu-doped CeO_2_	0	21.1
	Ni-doped CeO_2_	0	12.2
*F. oxysporum*	CeO_2_	0	15.7
	Fe-doped CeO_2_	0	23.1
	Cu-doped CeO_2_	0	19.4
	Ni-doped CeO_2_	0	10.3

## Conclusion

In conclusion, cubic CeO_2_ was doped successfully with Fe, Cu and Ni through co-precipitation method followed by calcination at 400°C for 4 h. The crystallite sizes were reduced from 9.76 to 3.46 nm by incorporating 3d transition metals into CeO_2_. Solid-state absorption analysis revealed a better spectral response and lower bandgap energy of Fe and Cu-doped CeO_2_ than unmodified CeO_2_ whereas increase in bandgap energy was noticed for Ni-doped CeO_2_. Moreover, the insertion of Cu and Fe into CeO_2_ suppressed its *e* ^*-*^–*h* ^*+*^ recombination process by generating trapping sites for photo-excitons. Among the synthesized photocatalysts in this study, Fe-doped CeO_2_ is excellent for the removal of 2-CP in the illumination of 70 W indoor LED white light.

## Data Availability

The original contributions presented in the study are included in the article/supplementary material, further inquiries can be directed to the corresponding authors.
